# Advanced Glycation End Products in Infant Formulas Do Not Contribute to Insulin Resistance Associated with Their Consumption

**DOI:** 10.1371/journal.pone.0053056

**Published:** 2013-01-02

**Authors:** Kristína Simon Klenovics, Peter Boor, Veronika Somoza, Peter Celec, Vincenzo Fogliano, Katarína Šebeková

**Affiliations:** 1 Department of Clinical and Experimental Pharmacotherapy, Medical Faculty, Slovak Medical University, Bratislava, Slovakia; 2 Institute of Physiology, Medical Faculty, Comenius University, Bratislava, Slovakia; 3 Institute of Molecular BioMedicine, Medical Faculty, Comenius University, Bratislava, Slovakia; 4 Division of Nephrology and Institute of Pathology, RWTH University of Aachen, Aachen, Germany; 5 German Research Center for Food Chemistry, Garching, Germany; 6 Department of Nutritional and Physiological Chemistry, University of Vienna, Vienna, Austria; 7 Department of Food Science, University of Naples Federico II, Naples, Italy; Wageningen University, The Netherlands

## Abstract

**Introduction:**

Infant formula-feeding is associated with reduced insulin sensitivity. In rodents and healthy humans, advanced glycation end product (AGE)-rich diets exert diabetogenic effects. In comparison with human breast-milk, infant formulas contain high amounts of AGEs. We assessed the role of AGEs in infant-formula-consumption-associated insulin resistance.

**Methods:**

Total plasma levels of N^ε^-(carboxymethyl)lysine (CML), AGEs-associated fluorescence (λ_ex_ = 370 nm/λ_em_ = 445 nm), soluble adhesion molecules, markers of micro- binflammation (hsCRP), oxidative stress (malondialdehyde, 8-isoprostanes) and leptinemia were determined, and correlated with insulin sensitivity in a cross-sectional study in 166 healthy term infants aged 3-to-14 months, subdivided according to feeding regimen (breast-milk- vs. infant formula-fed) and age (3-to-6-month-olds, 7-to-10-month-olds, and 11-to-14-month-old infants). Effects of the consumption of low- vs. high-CML-containing formulas were assessed. 36 infants aged 5.8±0.3 months were followed-up 7.5±0.3 months later.

**Results:**

Cross-sectional study: 3-to-6-month-olds and 7-to-10-month-old formula-fed infants presented higher total plasma CML levels and AGEs-associated fluorescence (p<0.01, both), while only the 3-to-6-month-olds displayed lower insulin sensitivity (p<0.01) than their breast-milk-fed counterparts. 3-to-6-month-olds fed low-CML-containing formulas presented lower total plasma CML levels (p<0.01), but similar insulin sensitivity compared to those on high-CML-containing formulas. Markers of oxidative stress and inflammation, levels of leptin and adhesion molecules did not differ significantly between the groups. Follow-up study: at initial investigation, the breast-milk-consuming infants displayed lower total plasma CML levels (p<0.01) and AGEs-associated fluorescence (p<0.05), but higher insulin sensitivity (p<0.05) than the formulas-consuming infants. At follow-up, the groups did not differ significantly in either determined parameter.

**Conclusions:**

In healthy term infants, high dietary load with CML does not play a pathophysiological role in the induction of infant formula-associated insulin resistance. Whether a high load of AGEs in early childhood affects postnatal programming remains to be elucidated.

## Introduction

In recent years, there has been a worldwide rise in the incidence of type 2 diabetes mellitus (T2DM) in children and adolescents, albeit T2DM was along back thought to be unique to adults [1]. The initial step in the development of T2DM is a decrease in insulin sensitivity, frequently associated with obesity [2]. In infants, the risk of insulin resistance is particularly high for newborns who are small for their gestational age and undergo rapid postnatal weight gain to obesity [3]. Much less attention is paid to decreased insulin sensitivity in infants appropriate for gestational age, although it has been well documented that formula feeding is associated with reduced insulin sensitivity and increased insulin secretion [4]–[6]. The composition of human breast milk differs profoundly from that of infant formulas, and to accommodate the infant’s needs best it changes dynamically during breast-feeding period [7]–[9]. Higher protein content, lower concentrations of long-chain polyunsaturated fatty acids, and presumably the lack of insulin-sensitizing hormones as well as numerous other biologically active substances in infant formulas in comparison with breast-milk, are thought to play a pathophysiological role in formula-feeding-associated decreased insulin sensitivity [10]–[12]. Recently, it has been suggested that food-derived advanced glycation end-products (AGEs) in AGE-rich infant formulas might precondition the infants to insulin resistance via induction of inflammation and oxidative stress [13].

Industrial processing of infant formulas requires heat-treatment. This results in the formation of substantial amounts of AGEs, which may exceed those present in human breast-milk up to 670-fold [14]–[17]. We have shown previously that AGEs from formulas are at least partially absorbed and contribute to the circulating pool of AGEs [14]. Vast majority of studies in rodents and in adult humans suggest that an excessive intake of highly thermally processed foods, rich in AGEs, may affect circulating AGE levels and may thus play a role in a wide range of harmful health effects, e.g. diabetogenic, pro-oxidative and pro-inflammatory events [13], [18]–[20]. However, some other studies evidenced beneficial effects, as reviewed in [21]. Interaction of AGEs with their specific cell surface receptors RAGE may result, among others, in the production of reactive oxygen species and induction of micro-inflammation, which may in turn accelerate the formation of AGEs, and exacerbate insulin resistance [2], [22], [23]. However, some studies have disputed these findings: there are still controversies on the identity of the actual AGEs that initiate the RAGE-mediated reactions, and questions regarding evidence that endotoxin contamination of AGE protein preparations compromised the results of some studies [24], [25]. Experimental studies show that AGEs *per se* may initiate the insulin-resistant state in skeletal muscle and adipocytes [26]–[28], or decrease the insulin content and secretion in pancreatic islets [29]–[31]. In line with the experimental data, a direct relationship between circulating AGE levels and insulin resistance was documented in non-obese non-diabetic adults [32]–[34].

Recent clinical data suggest that soluble RAGE (sRAGE) and vascular adhesion protein-1 (sVAP-1) may affect circulating AGE levels, and, thus, may play a role in insulin resistance. Circulating sRAGE bears the extracellular ligand binding domain but lacks the transmembrane and cytoplasmic domain. By removing or neutralizing circulating RAGE ligands, it acts as a natural competitive inhibitor of signal transducing metabolic pathways [35]. In humans, low sRAGE levels are associated with insulin resistance [36]–[38]. Endothelial VAP-1 represents an adhesion molecule, possessing the enzyme activity of semicarbazide-sensitive aminooxidase (SSAO) [39]. SSAO degrades primary amines into corresponding aldehydes, producing hydrogen peroxide and ammonia [40]. Reactive aldehydes are potent glycating agents, while hydrogen peroxide contributes to oxidative stress, thus may accelerate formation of AGEs. In diabetic rats, SSAO/VAP-1 imposes antidiabetic action via activation of glucose metabolism in adipocytes and muscle cells [41]. In humans, hyperglycemia induces a rise in circulating sVAP-1, which directly correlates with plasma AGE levels [42], [43].

The cross-sectional and follow-up study described in this work was aimed at elucidating whether formula-feeding-induced rise in circulating AGE levels may play a role in the induction of formula-feeding associated insulin resistance in healthy infants. Our data suggest that increased levels of circulating CML and/or the levels of AGE-associated fluorescence of plasma imposed by infant formulas consumption do not play a role in formula-consumption-associated insulin resistance, either directly or indirectly by induction of oxidative stress, micro-inflammation, hyperleptinemia, or decline in circulating sVAP-1 levels.

## Research Design and Methods

The study was carried out according to the Declaration of Helsinki, after the approval of the protocol by the Ethics Board (Slovak Medical University, Bratislava) and after obtaining the written informed consent from the mothers/legal guardians of the children.

The present cross-sectional study includes data from one hundred and sixty-six 3-to-14-month-old healthy term infants of Central European descent, infants of apparently healthy mothers residing in Bratislava and surroundings, examined from March 2006 to December 2008 in frames of ICARE (Impeding neoformed Contaminants Accumulation to Reduce their health Effects) study. From among 231 recruited 3–14-month-old infants, 65 met the exclusion criteria (pathology during physical examination, elevated inflammatory markers, acute/recurrent inflammatory or chronic diseases including allergies, positivity for antibodies against HCV/HIV, prematurity, being born small for their gestational age, and infant’s feeding regimen not compliant with Slovak recommendations for their age; and/or diagnosis of chronic disease or gestational diabetes in the mother) and were excluded from the present evaluation.

### Subjects

#### Cross-sectional study

**Figure 1:**
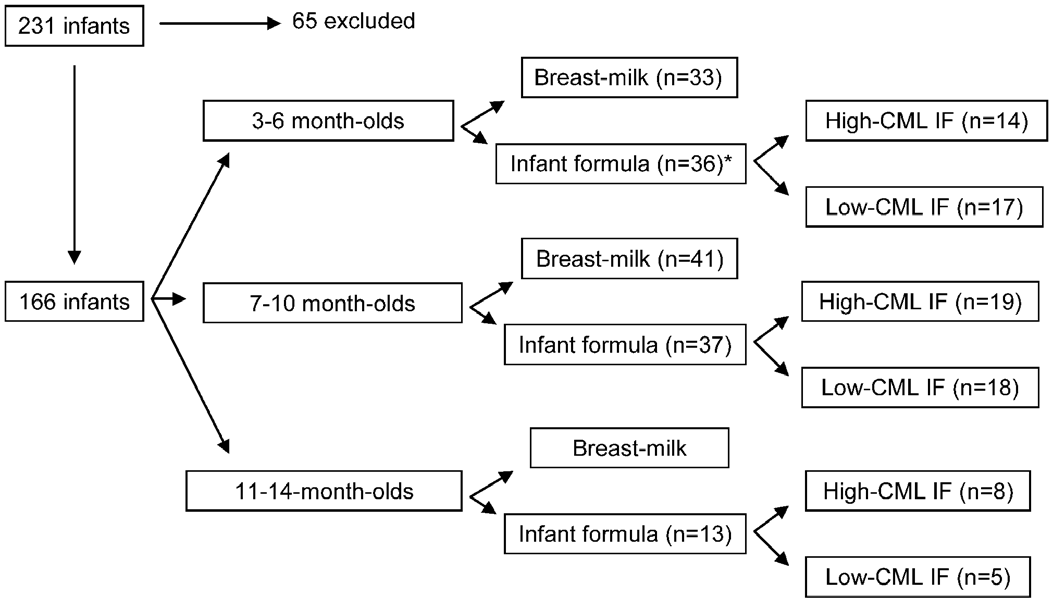
Recruitment and allocation of the infants according to age and feeding regimen. IF: infant formula; CML: N^ε^-(carboxymethyl)lysine; *: Five infants could not be uneuquivocally assigned to any group (four due to concurrent administration or just recent switch from low- to high-CML-containing formula, one child consumed formula not analyzed for CML content).

Cohort characteristics are given in Table 1. Infant's feeding regimen was recorded into the questionnaire filled in by mothers/legal guardians of the infants under supervision of pediatricians/educated nurses. If the child had received infant formula, the brand and the age at which formula feeding had started were recorded. Infants were allocated according to their age into 3 groups: 3-to-6-month-old infants (n = 69), 7-to-10-month-old weaning infants (n = 78), and older infants aged 11-to-14 months (n = 19). Each age-group was divided according to feeding regimen into breast-milk- and formula-receiving subgroup. Formula-fed infants consumed 17 different types of infant formulas. From among these, CML content was determined in 16 (marketed in Slovakia), as published elsewhere [14]. Based on these data [14], formula consuming groups were subdivided according to the CML content of the formulas. Formulas containing roughly 5-to-11 mg CML/100 g protein were considered as low CML-containing formulas, while those with 16-to-63 mg CML/100 g protein as high CML-containing formulas. Eight low CML-containing formulas were non-hydrolyzed and two were hydrolyzed formulations, while within the high CML-containing formulas one out of six was non-hydrolyzed [14]. Flow-chart depicting allocation of the infants according to age and feeding regimen is given in Figure 1.

**Table 1:** Cohort characteristics and blood chemistry data – cross-sectional study.

	3–6 month-olds	7–10 month-olds	11–14 month-olds
**Age (months)**			
BM	5.2±0.2	8.4±0.2	12.3±0.5
IF	5.0±0.1	8.3±0.2	12.1±0.4
Low-CML IF	4.8±0.2	8.0±0.3	11.5±0.4
High-CML IF	5.2±0.2	8.4±0.3	13.1±0.4^+^
**Gender (Female/Male)**			
BM	11/22	15/26	3/3
IF	17/19	20/17	7/6
Low-CML IF	6/11	10/8	3/2
High-CML IF	6/8	10/9	4/4
**Gestational age (weeks)**			
BM	39.6±0.2	39.2±0.2	39.4±0.4
IF	39.6±0.2	39.6±0.2	39.6±0.3
Low-CML IF	39.8±0.3	39.5±0.4	39.9±0.3
High-CML IF	39.3±0.4	39.6±0.3	39.0±0.7
**Birth weight (g)**			
BM	3632±76	3323±87	3252±275
IF	3143±89***	3305±80	3370±160
Low-CML IF	2937±122	3300±144	3308±241
High-CML IF	3227±136	3309±98	3470±178
**Body weight (g)**			
BM	7551±172	8417±179	9474±552
IF	6768±179**	7883±164*	9206±326
Low-CML IF	6581±278	7771±210	8981±456
High-CML IF	6859±270	7984±258	9567±440
**Mean daily weight gain (g)**			
BM	25±1	20±1	17±1
IF	25±1	19±1	16±1
Low-CML IF	26±1	20±1	17±1
High-CML IF	24±2	19±1	16±1
**Creatinine (µmol/l)**			
BM	30; 25–38	30; 27–35	27; 26–37
IF	27; 23–30*	29; 24–48	34; 26–48
Low-CML IF	26; 23–31	35; 24–49	34; 25–48
High-CML IF	28; 24–30	26; 24–33	27; 24–32
**AGE-Fl (AU)**			
BM	92±8	128±6^##^	161±19^##^
IF	123±6**	155±6**,##	162±9^##^
Low-CML IF	109±6*	149±9^##^	159±11^##^
High-CML IF	132±9**,+	158±8**	166±19
**sRAGE (pg/ml)**			
BM	1899±142	2076±131	2229±339
IF	1807±115	1904±119	1802±170
Low-CML IF	1838±196	1930±166	1721±302
High-CML IF	1840±181	1813±175	1852±216
**Glucose (µmol/l)**			
BM	4.6; 4.3–4.8	4.5; 4.1–4.9	4.1; 3.6–4.4
IF	4.1; 3.6–4.7**	4.4; 4.0–4.9	4.7; 4.3±5.0*,#
Low-CML IF	4.0; 3.6–5.1	4.4; 4.0–4.9	4.7; 4.5–5.3*
High-CML IF	4.4; 4.0–4.7	4.3; 3.8–4.8	4.4; 3.8–4.9
**Insulin (µIU/ml)**			
BM	2.8; 1.5–5.7	3.7; 2.4–5.1	2.4; 1.6–3.5
IF	8.1; 3.2–15.4***	4.1; 2.7–7.7	3.9; 1.7–9.4
Low-CML IF	9.0; 4.6–15.1**	4.5; 2.7–8.8	4.6; 1.5–9.0
High-CML IF	4.2; 2.5–11.3	3.6; 2.6–5.9	2.9; 1.9–15.4
**hsCRP (mg/l)**			
BM	0.2; 0.2–0.3	0.2; 0.2–0.3	0.2; 0.9–2.0
IF	0.2; 0.2–0.4	0.2; 0.2–0.7*	0.8; 0.2–2.3
Low-CML IF	0.2; 0.2–0.6	0.3; 0.2–1.9^+^	0.6; 0.2–3.9
High-CML IF	0.2; 0.2–0.4	0.2; 0.2–0.4	0.8; 0.5–2.3
**sICAM-1 (ng/ml)**			
BM	432±31	411±14	524±106
IF	419±26	414±21	435±13
Low-CML IF	377±25	363±17	446±14
High-CML IF	484±52	441±32	419±25
**sVCAM-1 (ng/ml)**			
BM	2263±215	1930±151	1436±252
IF	2093±175	1761±106	1431±144
Low-CML IF	1919±120	1631±103	1529±201
High-CML IF	2383±413	1847±183	1274±200
**sVAP-1 (ng/ml)**			
BM	542±22	555±20	580±65
IF	546±20	537±28	563±38
Low-CML IF	498±31	562±48	598±53
High-CML IF	579±29	520±33	506±46
**Leptin (ng/ml)**			
BM	3.1; 2.5–5.3	2.4; 1.8–3.0^##^	ND
IF	2.9; 2.4–4.0	2.1; 1.5–3.0^##^	ND
Low-CML IF	3.1; 2.7–3.9	2.3; 1.4–3.0	ND
High-CML IF	2.7; 2.0–5.1	1.9; 1.6–3.1	ND

Infants were allocated according to their age and feeding regimen. From among 69 infants 3-to-6-month-olds, 33 were exclusively breast-fed (BM) and 36 received infant formula (IF): 17 consumed formulas with high- and 14 with low-CML-content. Fourty-one 7-to-10-month-old infants were weaned from breast-milk, while 37 from formulas: 18 infants consumed low-CML- and 19 high-CML-containing formulas. Six older infants aged 11-to-14 months still received breast-milk as a supplement to diversified diet, while 13 drunk formula (5 consumed low-CML- and 8 high-CML-containing formulas). AGE-Fl: plasma advanced glycation end-products specific fluorescence; AU: arbitrary units; sRAGE: soluble receptor for advanced glycation end products; hsCRP: high-sensitive C-reactive protein; sICAM-1: soluble intercellular adhesion molecule-1; sVCAM-1: soluble vascular adhesion molecule-1; sVAP-1: soluble vascular adhesion protein-1;

* : p<0.05 vs. the corresponding age-group consuming breast-milk;

** : p<0.01 vs. the corresponding age-group consuming BM;

*** : p<0.001 vs. the corresponding age-group consuming BM;

+: p<0.05 vs. the corresponding age-group consuming infant formula with low-CML content;

#: p<0.05 vs. the 3-to-6-month-old infants on the same feeding regimen;

##: p<0.01 vs. the 3-to-6-month-old infants on the same feeding regimen; results are given as mean±sem or median and interquartile range;

ND: not determined.

In Slovakia, the recommendation is to not introduce solid foods before 6 month of age. Thus, 33 included 3-to-6-month-old infants were exclusively breast-fed. From among their 36 formula/mixed-fed counterparts, 27 were exclusively formula-fed, in 6 infants fruits or vegetables (raw or cooked) were added to one daily portion of formula, or a portion was replaced with this serving, and 3 were mixed-fed, i.e. received concurrently breast-milk and infant formula since their birth. The exclusion of mixed-fed infants, or those consuming fruits or vegetables, from the analysis did not alter the results (data not given).

In seventy-eight 7-to-10-month-old weaning infants, fruits, vegetables, flour, fish and lean meat were stepwise introduced to replace either breast-milk (n = 41), or infant formula (n = 37).

All 11-to-14 month-olds infants consumed a mixed diet. Six of them still received breast-milk (1–2-times/day) as a supplement to diversified diet, while 13 drunk formula (2–3-times/day).

#### Follow-up study

Thirty-six infants from the cross-sectional study, aged 5.8±0.3 months, were followed-up for another 7.5±0.3 months. Eleven out of them still received breast-milk as a supplement to the infant’s mixed diet at follow-up. Twenty-five infants were formula-fed at basal check-up, and received formulas as a supplement to infants’ mixed diet at follow-up. Only the initial sampling values of the infants participating in a follow-up study were included into the evaluation of the cross-sectional data. Cohort characteristics are given in Table 2.

**Table 2:** Characteristics of the followed-up infants.

	Basal data	Follow-up
**Gestational age (weeks)**		
BM	39.4±0.5	–
IF	39.4±0.4	–
**Birth weight (g)**		
BM	3562±125	–
IF	2956±113**	–
**Weight (g)**		
BM	7964±239	10549±295
IF	6879±228*	9440±286*
**Mean daily weight gain (g)**		
BM	22±1	16±1
IF	24±1	18±1
**Creatinine (µmol/l)**		
BM	28; 24–34	30; 28–35
IF	28; 24–31	27; 24–33
**Glucose (mmol/l)**		
BM	4.5; 4.2–4.9	4.3; 4.1–4.7
IF	4.0; 3.7–4.4*	4.2; 4.0–4.59
**Insulin (µIU/ml)**		
BM	3.1; 1.3–5.8	3.1; 2.6–5.1
IF	6.1; 3.8–10.9*	5.1; 3.1–7.8
**QUICKI**		
BM	0.425±0.022	0.411±0.020
IF	0.373±0.008*	0.385±0.009
**CML (ng/ml)**		
BM	711; 513–879	839; 624–1024^++^
IF	1218; 867–1404**	1129; 820–1427
**AGE-Fl (AU)**		
BM	101±13	171±9^++^
IF	132±10*	162±9
**sRAGE (pg/ml)**		
BM	1888±234	1897±152
IF	2034±143	1977±161
**hsCRP (mg/l)**		
BM	0.2; 0.2–0.4	0.2; 0.2–0.3
IF	0.2; 0.2–0.6	0.3; 0.2–0.6
**sICAM-1 (ng/ml)**		
BM	434±31	422±25
IF	403±23	441±31
**sVCAM-1 (ng/ml)**		
BM	2655±470	1924±394
IF	1922±90	1706±155
**sVAP-1 (ng/ml)**		
BM	530±33	266±42^++^
IF	539±32	296±21^++^

Thirty-six infants aged 5.8±0.3 (Basal data) months were followed-up 7.5±0.3 months later (Follow-up). BM: 11 infants exclusively breast-fed at basal sampling and receiving breast-milk as a supplement to infants’ mixed diet at follow-up; IF: 25 infants consuming infant formulas at basal investigation as well as at follow-up; QUICKI: Quantitative insulin-sensitivity check index; CML: plasma N^ε^-(carboxymethyl)lysine; AGE-fl: plasma advanced glycation end products specific fluorescence; sRAGE: soluble receptor for advanced glycation end products; hsCRP: high-sensitive C-reactive protein; sICAM-1: soluble intercellular adhesion molecule-1; sVCAM-1: soluble vascular adhesion molecule-1; sVAP-1: soluble vascular adhesion protein-1;

* : p<0.05 vs. the breast-milk consuming group at corresponding time interval;

** : p<0.01 vs. the breast-milk consuming group at corresponding time interval;

+: p<0.05 vs. the basal sampling in the same feeding regimen group;

++: p<0.001 vs. the basal sampling in the same feeding regimen group; results are given as mean±sem or median and interquartile range;

–: not applicable.

### Methods

Mother’s age, her actual weight, height, and weight gain during the pregnancy were recorded, and body mass index (BMI, kg/m^2^) was calculated.

Mothers were asked not to feed the infants 3 hours prior to blood sampling. If mother confirmed the child was fasting at least 3 hours, 4-to-5 ml of blood were collected from infant’s antecubital vein in the morning hours (between 7.30 and 9.30 a.m.). Plasma was obtained by standard centrifugation procedure. Hemolytic samples were excluded from analyses. Spot urine obtained on the day of blood sampling (around the time of blood collection) from 23 breast-milk- and 21 formula-fed 3-to-6-month-old infants was analyzed for 8-isoprostanes.

#### Standard blood and urine chemistry

Plasma glucose and creatinine (Vitros 250 analyzer, J&J, Rochester, USA) were determined. Commercial radioimmunoassay was used to analyze immunoreactive insulin (Immunotech, Prague, Czech Republic). Insulin sensitivity was evaluated by Quantitative insulin-sensitivity check index (QUICKI) [44].

#### Special analyses

Total CML concentration in plasma was determined using an ELISA assay (MicroCoat Biotechnologie GmbH, Bernried, Germany), after pretreatment of the samples with proteinase K (Roche, Mannheim, Germany) according to manufacturer’s instructions. ELISA kits, performed according to manufacturers’ instructions, were also used to determine plasma concentration of sRAGE (R&D, Minneapolis, MN, USA), high-sensitivity C-reactive protein (hsCRP, ImmunDiagnostik, Bensheim, Germany), leptin, soluble intercellular adhesion molecule-1 (sICAM-1), soluble vascular cell adhesion molecule-1 (sVCAM-1), sVAP-1, leptin (all BenderMedSystem Inc., Vienna, Austria), and urinary 8-isoprostanes (Cayman Chemicals, MI, USA). Plasma malondialdehyde (MDA) levels were determined employing HPLC method with fluorimetric detection [45], plasma AGE-associated fluorescence (λ_ex_ = 370 nm/λ_em_ = 445 nm) was determined [46]. For technical reasons (insufficient biological material), plasma MDA levels were determined only in 33 breast-milk- and 18 infant-formula-receiving infants, and plasma leptin levels were not determined in the 11-to-14-month-olds infants. Renal excretion of 8-isoprostanes was expressed as a ratio to urinary creatinine.

#### Statistical analyses

Sample size was calculated using OpenEpi statistical program on the assumption that 3-to-6-month-olds breast-milk- versus formula-fed infants differ in plasma AGE levels by 50%, and SD represents 50% of the mean [14]; with confidence interval 95%, power 80%, and sample size ratio 1, calculated sample size was 16. Normally distributed data are given as mean ± SEM, those not fitting to normal distribution as median, and interquartile range. Two-sided Student’s t-test (paired or unpaired, as appropriate), or Wilcoxon and Mann-Whitney U-test were used to compare 2 sets of data, as appropriate. Three sets of data (fitting to normal distribution) were compared using ANOVA with Scheffe’s post-hoc test. Data displaying non-parametric distribution were compared using Kruskal-Wallis test with post-hoc Mann-Whitney U-test with Bonferonni correction for target alpha. Categorical data were compared using *chí-*square. Pearson’s (indicated in results as “r”) or Spearman’s (if the correlated data did not fit to normal distribution) correlation coefficients were calculated. Multiple regression analyses were performed using General linear model (GLM). P<0.05 was considered significant. For evaluation, SPSS v. 16 statistical program was used.

## Results

The mothers of the breast- and formula-fed infants did not differ significantly by age, BMI, and the weight gain during the pregnancy (Table 3).

**Table 3:** Characteristics of the mothers of included infants.

	Infants’ age		
	3–6 month-olds	7–10 month-olds	11–14 month-olds
**Age (years)**			
BM	30.4±1.0	28.7±0.7	28.6±1.1
IF	29.6±1.4	28.6±1.3	32.8±2.8
**BMI (kg/m^2^)**			
BM	23.4±0.7	22.6±4.9	22.3±0.8
IF	26.5±1.7	22.9±0.9	25.7±1.6
**Pregnancy weight gain (kg)**			
BM	13.8±1.1	14.0±0.8	14.3±3.5
IF	14.4±1.1	14.4±1.4	13.5±2.0

BM: mothers of exclusively breast-fed infants; IF: mothers of infant formulas recieving infants; BMI: body mass index.

### Cross-sectional Study

#### Basic cohort characteristics

Anthropometric data of the infants at birth and at investigation are presented in Table 1. Within the 3 age-groups, infants receiving breast-milk did not differ significantly from those receiving formulas by age, gestational age, mean daily weight gain, and plasma creatinine concentration. Proportion of girls and boys in the breast-milk- and formula-fed groups of corresponding age did not differ significantly (chi square: n.s., all). Formula-fed 3-to-6-month-old infants had lower birth weight and body weight at investigation when compared with their breast-milk-fed counterparts. Although the mean birth weights of the cohorts differed significantly, they were within the “normal” birth weight range of eutrophic term-infants (2500–4000 g). The same holds true for the age-adjusted body weight at investigation. Formula-fed 7-to-10-month-olds displayed similar birth weight, but lower body weight if compared with the breast-milk-fed infants. Again, the means differed significantly, despite that the age-adjusted body weights were within the normal range. 11-to-14-month-old breast-milk- and formula-fed infants did not differ by the birth and body weight significantly.

Infants consuming low- vs. high-CML-containing formulas did not differ significantly in either characteristics, except for the fact that 11-to-14-month-olds infants consuming low-CML-containing formulas were younger (by 6 weeks in mean, p = 0.03) than those on high-CML-containing formulas (Table 1).

#### Theoretical daily dietary CML burden

From among 56 breast-milk samples analyzed for CML content [14], 21 were donated by the mothers of 3-to-6-month-old herein evaluated infants. The calculated daily burden of CML in the 3-to-6-month-old breast-fed infants did not exceed 0.03 mg CML/day, thus was less than 0.004 mg CML/kg body weight/day. From among 36 formula-fed 3-to-6-month-old infants, 17 consumed formulas with high-CML content and 14 were fed low-CML-containing formulas (Table 1). Five infants could not be unequivocally assigned to any group (concurrent administration/recent switch from low- to high-CML-containing formula, consumption of formula not analyzed for CML content). The theoretical daily burden of CML ingested from formulas, calculated on the basis of determined CML content of formula [14] and daily dosage specified by the manufacturers, ranged from 8.8 mg/day to 10.3 mg/day in infants consuming high-CML-containing formulas, while those on low-CML-containing formulas ingested amounts from 1.8 mg/day to 2.2 mg/day. Taking into account the estimated mean body weight at the time of investigation, infants on low-CML-containing formulas ingested daily 0.27–0.33 mg CML/kg body weight, e.g. up-to 83-fold higher (0.33 mg/kg body weight/day) amounts in comparison with their breast-milk-fed counterparts. Those on high-CML-containing formulas consumed 1.3–1.5 mg CML/kg body weight/day, thus even 375-fold higher amounts of CML/kg/day than the corresponding breast-milk-fed infants.

As calculated on the basis of 19 breast-milk samples obtained from the mothers of 7-to-10-month-old infants, the maximal amounts of ingested CML corresponded to those in 3-to-6-month-olds. Eighteen 7-to-10-month-old infants were administered formulas with low-CML contents, and 19 received high-CML-containing formulas (Table 1). The calculated mean daily burden of CML arising from low-CML-containing formulas ranged between 1.0 mg/d to 1.3 mg/day, i.e. 0.13–0.17 mg CML/kg body weight/day (up-to 43-fold higher than the corresponding breast-milk-fed infants), while consumption of high-CML-containing formulas represented an intake between 3.7 mg and 6.4 mg CML/day, corresponding to 0.46–0.80 mg CML/kg body weight/day (e.g. up-to 200-fold more CML per kg body weight in comparison with breast-milk fed counterparts).

Five 11-to14-month-old infants received high-CML-containing formulas as a supplement to a mixed infant diet, while 8 consumed low-CML formulas. Since the manufacturers do not indicate the dosage of formula for this age-group, and the breast-milk from mothers of 11-to-14-month-old infants was not available, the theoretical CML dietary burden coming from the milk drunken was not calculated. In any case, the vast majority (i.e. 97%) of ingested AGEs in this age-group does come from the solid foods [13] in which CML content was not determined in present study.

#### CML

**Figure 2:**
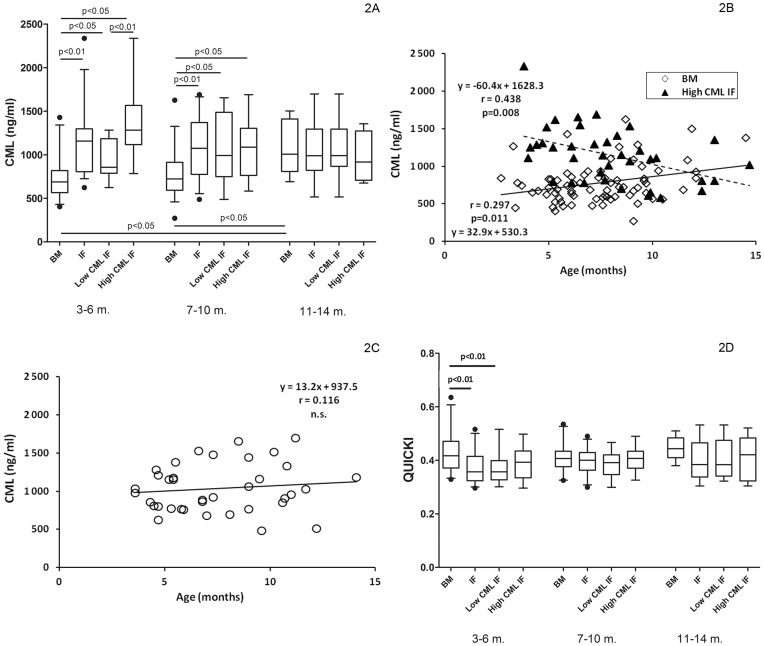
Total plasma N^ε^-(carboxymethyl)lysine (CML) levels and insulin sensitivity in breast-milk- and formula-fed infants included in the cross-sectional study. 2A/Plasma total CML concentration in relation to feeding regimen and age. Box-plots represent median, interquartile range and 5^th^–95^th^ percentile; 2B/Age-dependence of plasma total CML concentration in the breast-milk-fed infants (BM) and those consuming high-CML-containing infant formulas (IF); 2C/Relationship between plasma total CML concentration and age in the low-CML-containing infant formulas fed healthy infants; 2D/Insulin sensitivity assessed by Quantitative insulin-sensitivity check index (QUICKI) in the breast-milk- (BM) and infant formula-fed (IF) infants, low-CML-containing formulas (Low-CML IF) and high-CML-containing formulas (High-CML IF) consuming infants aged 3-to-6-months (m.), 7-to-10-months, and 11-to-14-months. Box-plots represent median, interquartile range and 5^th^–95^th^ percentile.

Compared with breast-fed infants, 3-to-6-month-olds and 7-to-10-month-old formula-fed infants displayed higher plasma total CML concentrations (Figure 2a). Total CML levels were higher in the 11-to-14-month-old breast-milk fed infants if compared with the 2 younger breast-milk-fed groups. Since the birth weight and/or body weight of the formula- and breast-fed 3-to-6-month-old and 7-to-10-month-old infants differed significantly, multivariate analysis was employed to elucidate whether the weights independently and significantly affected the total CML levels. GLM confirmed that feeding regimen was a single independent significantly contributing factor (data not given). Total CML levels showed an age-dependent rise if all breast-milk-consuming infants were evaluated together (Fig. 2b).

3-to-6-month-olds on the low-CML-containing formulas presented significantly lower total plasma CML levels (Figure 2a) in comparison with the group fed by high-CML-containing formulas. Total plasma CML levels were higher in the 3-to-6-month-olds and 7-to-10-month-olds on low-CML-as well as high-CML-containing formulas than in the corresponding breast-milk-fed infants (Figure 2a). While in the infants fed high-CML-containing formulas, total plasma CML concentration decreased by age (Fig. 2b), no significant relationship was observed in those on low-CML-containing formulas (Fig. 2c).

#### Plasma AGE-associated fluorescence (λ_ex_ = 370 nm/λ_em_ = 445 nm)

3-to-6-month-olds and 7-to-10-month-old formula-fed infants displayed higher plasma fluorescence than their breast-milk-fed counterparts (Table 1). It showed an age-dependent rise if either all breast-milk-consuming infants (y = 9.09x+47.45, r = 0. 547, p = 0.001), or the formula-fed infants (y = 5.68x+100.24, r = 0.446, p = 0.001) were evaluated together.

3-to-6-month-olds on the low-CML-containing formulas presented significantly lower plasma fluorescence (Table 1) in comparison with the group fed by high-CML-containing formulas.

#### Insulin sensitivity

Breast-milk-fed 3-to-6-month-old infants presented higher plasma glucose concentration in comparison with their formula-fed counterparts but they maintained glycemia with lower insulin levels (Table 1), resulting in higher insulin sensitivity (Figure 2d). In the 7-to-10-month-olds and 11-to-14-month-olds glycemia, insulinemia and insulin sensitivity did not differ significantly between the breast-milk- and formula-consuming groups. Correction for the birth or body weight did not alter the results, and General linear model did not indicate these parameters as independent significant contributors (data not given). However, correction for mean daily weight gain revealed that both, feeding regimen (p<0.001) and mean daily weight gain (p = 0.003) significantly affect insulin sensitivity (corrected model: p<0.001), contributing to 12.2% of its variability. In the whole cohort insulin sensitivity displayed significant inverse relationship to mean daily weight gain (y = −0.002x+0.453, r = −0.200, p = 0.014). This relationship was on the account of the formula-fed infants (y = −0.003x+0.452, r = −0.333, p = 0.003), since the correlation in the breast-milk-fed cohort was not significant (data not given). When the 3 age-groups were analyzed separately, GLM indicated concurrent significant independent impact of feeding regimen (p<0.001) and the mean daily weight gain (p = 0.003) only in the 3-to-6-month olds (corrected model: p<0.001; R^2^: 0.266). In this age group the simple correlation between insulin sensitivity and the mean daily weight gain was inverse both in the breast- (r = −0.385, p = 0.033) and formula-fed-infants (r = −0.387, p = 0.026). No significant relationship between QUICKI and total plasma CML or AGEs-associated fluorescence was revealed in either group, and total plasma CML or AGEs-associated fluorescence showed no impact on insulinemia or insulin sensitivity in multiple correlation model (data not shown).

Infants consuming low- versus high-CML-containing formulas did not differ significantly either by plasma glucose, insulin (Table 1), or insulin sensitivity (Figure 2d) in either age-group. 3-to-6-month-old infants consuming low- (but not high-) CML-containing formulas displayed higher concentrations of insulin and lower insulin sensitivity if compared with the breast-milk-fed counterparts (Table 1 and Figure 2d).

#### Soluble RAGE

No significant difference was revealed between the breast-milk- and formula-consuming infants in either age-group (Table 1). In the formula-consuming group plasma sRAGE levels correlated directly with those of total CML (y = 0.13x+863.7, r = 0.243, p = 0.031).

Consumption of high- or low-CML-containing infant formulas was not reflected by significant difference in sRAGE levels (Table 1). In the infants consuming high-CML-containing formulas plasma sRAGE levels showed direct relationship to total CML (y = 0.072x+1051.4, r = 0.337, p = 0.024).

#### hsCRP and adhesion molecules

The breast-milk- and formula-consuming infants did not differ significantly by hsCRP, sICAM-1, sVCAM-1 and sVAP-1 levels in either age-group (Table 1). No significant relationship between total plasma CML, glycemia, insulinemia or QUICKI, and sVAP-1 was revealed in either feeding-regimen group (data not given). However, sVAP-1 levels correlated directly with those of sRAGE in the both cohorts (mother-milk-fed: y = 0.052x+446.4, r = 0.337, p = 0.002; formula-fed: y = 0.063x+427.9, r = 0.300, p = 0.006).

Infants consuming low-CML-containing formulas did not differ significantly from those fed high-CML-containing formulas by plasma hsCRP, sICAM-1, sVCAM-1 and sVAP-1 levels (Table 1). No significant relationship between total plasma CML, glycemia, insulinemia or QUICKI, and sVAP-1 levels was revealed in either group (data not given). sVAP-1 and sRAGE levels correlated directly in both cohorts (low-CML-containing formula-fed group: y = 0.091x+376.5, r = 0.386, p = 0.044; high-CML-containing formula-fed group: y = 0.052x+443.5, r = 0.326, p = 0.045).

#### Oxidative stress markers

In the 3-to-6-month-old infants, plasma MDA levels did not differ significantly between the groups: breast-milk-fed: 1.3±0.1 µmol/l; formula-fed: 1.1±0.1 µmol/l; p = 0.08. Correspondingly, no significant difference in urinary excretion of 8-isoprostanes was revealed: breast-milk-fed: 52±4 ng/mmol creatinine; formula-fed: 54±4 ng/mmol creatinine; p = 0.68.

#### Leptin

The breast-milk- and formula-consuming infants did not differ significantly in leptinemia (Table 1). However, in both, the breast-milk- and formulas-consuming groups, 7-to-10-month-olds infants displayed lower leptin concentrations than the 3-to-6-month olds. To elucidate whether this decline was associated with ageing or change in body weight, GLM was performed entering the feeding regimen as fixed factor and age and mean daily weight gain as covariates. Mean daily weight gain appeared as a single significant independent contributor (corrected model: p<0.001; R^2^: 0.332). This relationship was confirmed in the whole cohort by a single regression model (y = 216x−1650; r = 0.577, p<0.001). Correlation remained significant even if the breast- and formula-fed infants were evaluated separately (r = 0.617, and r = 0.571, respectively; both: p<0.001). No significant relationship between insulin, QUICKI, total plasma CML or AGEs-associated fluorescence and leptin levels was revealed in either feeding-regimen group (data not shown).

Infants consuming low- vs. high-CML-containing formulas did not differ significantly by plasma leptin levels in either age-group (Table 1).

#### Effects of birth weight or actual body weight

Multivariate analysis (GLM) did not indicate any significant independent impact of birth weight or body weight on plasma AGE-associated fluorescence, sRAGE, markers of inflammation, oxidative stress, or leptin levels (data not given).

#### Effect of mothers’ weight gain during pregnancy

Neither of determined blood chemistry parameters in the infants displayed significant relationship with the mothers’ weight gain during her pregnancy either in the simple or in multiple regression models (data not given).

### Follow-up Study

#### CML

At baseline, the breast-milk-fed infants had significantly lower total plasma CML levels than the formula-receiving group. At the end of the follow-up period, no significant differences were observed between the groups: in the breast-milk-consuming infants the total plasma CML levels were higher than at baseline (Table 2).

#### Plasma AGEs-associated fluorescence (λ_ex_ = 370 nm/λ_em_ = 445 nm)

At baseline, but not at follow up, the breast-milk-fed infants displayed significantly lower plasma AGEs-associated fluorescence than the formula-receiving group. In the breast-milk-consuming infants the baseline plasma fluorescence was lower than at follow-up (Table 2).

#### Insulin sensitivity

At baseline, formula-fed infants displayed significantly lower glycemia and higher insulinemia, resulting in lower insulin sensitivity if compared with their breast-milk-fed counterparts. Neither of these parameters differed significantly at follow-up (Table 2).

#### Soluble RAGE

No significant difference was revealed between the breast-milk- and formula-consuming infants (Table 2).

#### hsCRP and adhesion molecules

The breast-milk- and formula-fed infants did not differ significantly by hsCRP, sICAM-1, sVCAM-1 and sVAP-1 levels (Table 2). In both cohorts the circulating sVAP-1 levels were significantly lower at follow-up in comparison with the baseline data. In the formula-consuming infants a direct relationship between sRAGE and sVAP-1 was revealed (y = 0.034x+269.5, r = 0.303, p = 0.034).

#### Leptin

In correspondence with the cross-sectional study data, the breast-milk- and formula-fed infants did not differ significantly by baseline leptinemia (3.2; 2.2–5.7 ng/ml, and 2.7; 1.8–3.8 ng/ml, respectively).

## Discussion

Breast-milk- versus formula-feeding represents a unique long-term human model of AGE-poor versus AGE-rich diets consumption, in which the adherence to the diet is doubtless. We show that, in healthy term infants, high dietary intake of AGEs in form of infant formulas is reflected by a rise in circulating total plasma CML levels, and AGEs-associated fluorescence, but it is neither involved in formula-consumption-associated reduced insulin sensitivity, nor accompanied by enhanced oxidative stress and micro-inflammation. Trends in total plasma levels of CML and AGE-associated fluorescence seem not to be the same over time.

### Plasma CML Levels

Lactose, a main milk sugar in mammals, is a potent glycating agent modifying lysine residues into CML and other Maillard reaction products [47]. In our study milk prepared from infant formulas contained 5.3%–8.3% lactose, 6.6% in mean. Human breast milk contains 6.9–7.2% lactose [48]. Thus, we postulate that the amount of ingested lactose in form of infant formula was similar to that ingested in breast milk; and the difference in lactose intake could not underlie observed differences in total plasma CML levels between the formula- and breast-fed infants.

Mericq et al. showed that in comparison with exclusively breast-fed newborns, in 12-month-olds on a mixed diet the daily intake of AGEs is 7.5-fold higher, reflected by an age-dependent rise in serum CML [13]. We confirmed this data: in infants weaned from breast-milk total plasma CML levels rise age-dependently, reflecting an increased dietary CML load imposed by a mixed diet. However, introduction of a mixed diet to high-CML-containing formulas-consuming infants is associated with an age-dependent decline in total plasma CML concentrations, implying that mixed diet imposes a lower dietary CML-load than formula consumption. Replacement of low-CML-containing formula with a mixed diet is not associated with apparent changes in circulating CML levels, indicating that the diet provides a similar oral CML-load to that of low-CML-containing formulas. When consuming a mixed diet, formerly breast-milk- and formula-fed infants present comparable total plasma CML levels.

Oxidative stress and inflammation may also lead to AGE formation [13], [49]–[51]. Our data do not suggest that these mechanisms contribute to elevation of circulating CML and AGEs-associated fluorescence in formula-consuming infants, since these infants neither present increased levels of markers of lipid peroxidation (MDA, 8-isoprostanes), nor of microinflammation (hsCRP, adhesion molecules), if compared with their breast-milk-fed counterparts.

In adults, an increase in serum SSAO/VAP-1 is mirrored by a rise in circulating AGEs, and oxidative stress markers [43]. Formula-fed infants neither present alterations in circulating levels of sVAP-1 and oxidative stress markers, nor significant correlation between plasma AGE and sVAP-1 levels. Thus, SSAO pathway is probably not involved in formula-consumption-associated rise in plasma AGE levels.

Exogenously administered sRAGE blocks the harmful effects of AGEs in animals by acting as a decoy receptor [52]. Endogenous sRAGE probably does not exert the same biological effect since serum levels of sRAGE in humans are 1000-fold lower than needed for binding to AGEs [52], and in healthy adults circulating AGEs positively, rather than inversely, correlate with sRAGE levels [53]. Although groups on different dietary regimen did not differ significantly in sRAGE levels, formula-consuming infants, particularly those administered high-CML-containing formulas, presented a positive relationship between total plasma CML and sRAGE concentrations, probably reflecting the enhanced cleavage of sRAGE from the cell surface RAGE. Correlation between sRAGE and sVAP-1 levels, both shed from cell surface by matrix metalloproteinases [41], [54], supports this assumption. Our data suggest that under long-term high-AGE diet endogenous sRAGE is not the determinant of total circulating CML levels.

Thus, during the period when milk represents a sole or major source of nutrition absorbed dietary infant formulas-derived AGEs account for elevated total plasma CML and AGE-associated fluorescence levels.

### Insulin Sensitivity

Exclusively breast-fed infants presented higher insulin sensitivity, and lower total plasma CML levels and plasma fluorescence than their formulas-consuming counterparts. This could support the presumption that an AGE-rich-diet-induced rise in AGEs might be involved in the induction of insulin resistance. However, several lines of evidence point against this assumption. First, in comparison with low-CML-containing-formulas, consumption of high-CML-containing formulas resulting in higher total circulating CML levels does not aggravate insulin resistance. Second, 7-to-10-month-olds weaned either from formula or breast-milk presented comparable insulin sensitivity, despite approximately 40-to-200-fold difference in the dietary CML burden imposed, reflected accordingly by higher total plasma CML levels in the formula-consuming group. Third, the introduction of a mixed diet, with substantially higher AGE contents than that in breast-milk [13], to exclusively breast-fed infants was not associated with a decrease in insulin sensitivity in our study. Fourth, in contrast to data from healthy adults [32]–[34], we did not reveal any significant relationship between total plasma CML levels or plasma AGEs-associated fluorescence and insulin sensitivity. A recent well-controlled study in healthy young adults also documented that ingestion of highly thermally processed food, in comparison with steamed diet, results in rise in plasma CML levels and decline of insulin sensitivity, without any correlation between these two parameters [19]. Although Mericq et al. [13] demonstrated a concomitant rise in plasma AGE levels and insulin resistance in healthy infants weaned from mother milk, no correlation between the two parameters was presented, preventing a direct comparison of our data with their findings. Fifth, in contrast to studies in adults, we did not observe an induction of oxidative stress- and microinflammatory-markers, which could contribute to the induction of insulin resistance [13], [49] during formula-feeding. Sixth, since high AGE-diet did not affect sVAP-1 levels, insulin resistance could not be elicited by sVAP-1 decline. VAP-1 has been found to have an antidiabetic function in the rats [41], and might contribute to lactation-associated insulin sensitivity in humans [55]. Seventh, the formula-feeding-induced rise in total plasma CML and AGEs-associated fluorescence was not accompanied by hyperleptinemia, which could participate in the induction of insulin resistance [56], [57]. Similar leptinemia between the breast-milk- and formula-fed infants was reported also by others [58]. And eighth, markers of oxidative stress and inflammation neither differed significantly between formula- and mother-milk-consuming infants, nor showed significant relationship to total plasma CML, or to insulin sensitivity. This contradicts the suggestion that formula-derived AGEs might precondition the infants to insulin resistance via induction of inflammation and oxidative stress [13]. Eventually, in our study either unspecified AGEs present in infant formulas possessing an antioxidant capacity, or fortification of formulas with antioxidant vitamins counteracted the AGEs-induced oxidative stress. High levels of antioxidant vitamins seem to exert similar protective effects in vegetarians, who, despite of high plasma AGE levels, present higher insulin sensitivity than omnivores [59].

A rapid growth in early childhood in infants born small for gestational age is considered as a risk factor for development of insulin resistance and metabolic syndrome in later life [3], [60]. However, Kerkhof et al. recently suggested that a gain in weight during the first 3 months of life, not the birth weight per se, was the most important determinant of prevalence of metabolic syndrome, and its signs, in young adults [12]. Our data showing that mean daily weight gains in 3-to-6-month-olds show inverse relation to insulin sensitivity regardless of the feeding regimen, are in line with the above mentioned observation, although Kerkhof et al. [12] did not investigate the effects of breast- versus formula-feeding. Our data also suggest that in the case of formula feeding this effect might not be restricted to early infancy, as indicated by inverse relationship between the mean weight gain and insulin resistance in infant formula-fed group generally. In addition, we revealed a tight relationship between leptin levels (a regulator of food intake and energy metabolism, [56]) and mean daily weight gain. Changes in circulating leptin levels are thought to be related to insulin-mediated glucose metabolism in adipose tissue [57]. In light of these facts the associations observed in our study in healthy, term and appropriate for gestational age infants require attention, and their potential clinical relevance remains to be confirmed in larger and prospective studies.

Although industrial heat-processing of infant formulas results in a substantial rise of early and advanced glycation end-products [17], in contrast to thermally processed foods consumed by adults, infant formulas generally contain only traces of, if any, other heat-born toxic substances [61]–[63]. Thus, as far as the ongoing debate whether dietary AGEs are harmful to human health or not is concerned [18], [21], [49], [50], [64], our data favor the assumption that the negative health effects of thermally processed foods do not result from ingestion of CML *per se,* but rather from other AGEs, or additive effects of different heat-processing-derived substances.

Relatively high proportion of the formula-fed infants was administered high-CML-containing (e.g. hypoallergenic, hydrolyzed) formulas, despite that food or other allergy was an exclusion criterion. In so far these formulas were not administered therapeutically. The intentional prescription of hydrolyzed formulas to infants with lower normal birth weight can be excluded, since the infants on high-CML-containing formulas tended to have higher birth weight. However, the high proportion of infants on hydrolyzed formulas did not stem purely from implementation of preventive measures, often applied if a sibling suffered from allergy. Some infants were shifted from non-hydrolyzed to hydrolyzed formulas if they did not accept the new formula well, or did not thrive well, e.g. during weaning or introduction of follow-on formula. In light of rising prevalence of allergies [65], [66] mothers are often convinced that hydrolyzed formulas are better, thus feed their baby with these despite being explained by the pediatrician that it is not indicated.

### Limitations

Some of the age-matched groups of formula- and breast-milk-fed infants differed by the body weight, due to the higher birth weights of the breast-fed infants. We suppose that this was a coincidence, since the infants were not randomly assigned to feeding-regimen groups. Other studies reporting higher birth weights of the breast-fed infants suggest that mothers who intend to breast feed are more conscious of their lifestyle during pregnancy, resulting in a higher birth weight of their child [67], [68]. Sample size was calculated to detect 50% difference in plasma AGEs in 3-to-6-month-olds infants, and thus could be underpowered to assess significant differences in some other studied parameters. Blood chemistry parameters were not analyzed after overnight fasting. In infants, the duration of fasting should be selected as a compromise between avoiding post-prandial blood sampling and obtaining a reasonable fasting interval not harming/endangering the child. The fasting time employed in our study (3 hours) fits well with that applied in other studies on insulin sensitivity in toddlers, e.g. fasting intervals of 2-to-4 hours [6], [69]. Exact duration of fasting prior to blood sampling was not recorded, thus the data could not be corrected for this parameter. Malondialdehyde, a widely used marker of oxidative stress [13], [32], [33], was determined only in 3-to-6-month-olds, not allowing the detailed analysis of the relationships to plasma AGE levels and markers of microinflammation. Although the total interference of low-molecular-weight fluorophores naturally occurring in plasma under physiological conditions with plasma fluorescence (λ_ex_ = 370 nm/λ_em_ = 445 nm) is low (up to 1%) [46], the interference of non-AGE-modified proteins cannot be excluded. The daily burden of ingested CML was not expressed upon determination of CML content in individual samples taken from each child’s formula. Planning the study we considered this approach, but we concluded that analysis of CML in individual formulas collected from each child’s household could bias the results substantially, since the collection of powder formula samples, their handling and storage between sampling in each household and hand-over in our laboratory, as well as the storage period prior to sending the samples for CML analyses to central laboratory (Garching, Germany in frames of ICARE project) could not be adequately standardized. Thus, we gave a priority to the approach described previously [14]: the formulas consumed by the enrolled infants were purchased in the pharmacy and analyzed for CML content, also assuming a standardized procedure for the processing of each formula which in general is aimed at low intra-individual variances. Our results are pertinent to the consumption of 17 different infant formulas produced by 5 different world renowned companies. It might not be excluded that other infant formulas contain different amounts of CML, or exert different effects on parameters determined herein. However, our data on the CML content of infant formulas, as well as the estimated difference in CML content between mother milk and infant formula, are in excellent accordance with those reported by Delatour et al. [14], [16]. It might be argued that inclusion of infants consuming 17 different formulas might have confounded the results. Taking into account that human milk composition changes during the lactation, and displays particular inter-individual differences, our formula-fed infants might represent even more homogenous group than the breast-milk-fed infants. We determined a single chemically defined AGE – CML. Data on CML urinary excretion are not presented, since CML levels in random spot urines neither reflect accurately the daily CML-load, nor can be used for calculation of dietary CML exposure. In the pilot study we showed that infant formula-fed infants excrete roughly 60-fold more CML than their breast-milk-fed counterparts [14]. In non-proteinuric subjects free CML adducts account for urinary CML excretion. Extremely large inter-individual variability of CML in random spot urines from formula-fed infants suggests a rapid elimination after the absorption [14]. This fits with the experimental data showing that the half-life of i.v. administered labeled fluorobenzylated-CML is very short (about 20 min) [70]. However, the biodistribution and elimination profile of this i.v. administered derivatized free adduct may differ from that of underivatized CML administered orally. At least but not at last, pre-pregnancy BMI of the mothers (not recorded in our study) could adversely affect the insulin sensitivity of the infant.

Taken together, our data support our previous hypothesis that high circulating AGEs in formula-consuming infants arise from high dietary intake of AGEs [14], and are in line with results from a recent study of Mericq et al. [13]. We confirmed that formula feeding is associated with the development of transient insulin resistance. In healthy infants, AGEs absorbed from infant formulas do not play a role in formula-consumption-associated insulin resistance, either directly or indirectly by induction of oxidative stress, micro-inflammation, hyperleptinemia or decline in sVAP levels. It remains to be elucidated whether the formula-feeding-associated high dietary load with AGEs in early infancy could exert negative health effects in vulnerable, sensitive, or diseased children; or predispose them to earlier, accelerated, or more serious manifestation of chronic degenerative diseases in later life.
